# Supplementary material for: Increased Waist Circumference after One-Year Is Associated with Poor Chewing Status
Source: Healthcare (Basel). 2024 Jul 5;12(13):1341. doi: 10.3390/healthcare12131341 (PMC11240897; doi:10.3390/healthcare12131341)
Supplement: Supplementary file 1 [file healthcare-12-01341-s001.zip › healthcare-3046926-supplementary.pdf]

Supplemental Table S1. Crude ORs and 95% CIs for an increase in WC of ≥ 5 cm after 1 year.

Factor		ORs	95% Cls	*p*-value
Beef/pork	Not daily intake	1	(reference)	0.461
	Daily intake	1.076	0.886-1.307	
Poultry	Not daily intake	1	(reference)	0.178
	Daily intake	1.187	0.925-1.524	
Fish	Not daily intake	1	(reference)	0.924
	Daily intake	1.015	0.751-1.371	
Dairy products	Not daily intake	1	(reference)	0.297
	Daily intake	1.092	0.925-1.289	
Sweet foods	Not daily intake	1	(reference)	0.222
	Daily intake	1.112	0.938-1.317	
Vegetables	Not daily intake	1	(reference)	0.297
	Daily intake	1.105	0.914-1.337	

Abbreviations: WC, waist circumference; ORs, odds ratios; CIs, confidence intervals.

* $p < 0.05$, using univariate logistic regression analysis.
